# Remarks on Follicular Enteritis, Occurring as a Complication of Continued Fever

**Published:** 1848-01

**Authors:** 


### BUFFALO MEDICAL JOURNAL

AND

### MONTHLY REVIEW.

**VOL. 3.** JANUARY, 1848. **NO. 8**

### ORIGINAL COMMUNICATIONS.

ART. I.—*Remarks on Follicular Enteritis, occurring as a complication of continued Fever. Continued from page* 385.

We concluded our remarks in the last No. by inquiring, if the fact of follicular enteritis being not a constant, but frequent complication, furnishes an occasion for sub-dividing continued fever into distinct varieties or species. This question has grown out of the investigation of the intestinal affection since the publication of Louis. So far as we know, Dr. Lombard was the first to advocate the affirmative to this question. Finding that the fevers of Great Britain did not uniformly present the "anatomical characteristic" of the fevers of Paris, he arrived at the conclusion that the former consist of two kinds, radically distinct from each other, the chief radical distinction, consisting in the presence, or absence of the intestinal lesions. A similar opinion has been advanced by Dr. Gerhard, who communicated some observations, made by him at the Pennsylvania hospital, in 1836, [Am. Jour. Med. Sciences, 1847] relating to a form of fever different from the fever endemial in Philadelphia and vicinity, evidently infectious, and traceable to emigrants newly arrived from Liverpool, England. In fifty autopsies of subjects dead with that form of fever, follicular enteritis was found but in one instance, and in that instance there had been some doubt as to the correctness of the diagnosis. From these and some other similar facts, taken in connection with other circumstances, a division has been instituted into two forms of fever, generally distinguished as typhus and *typhoid;* the latter supposed to present follicular enteritis as an invariable complication, the former as invariably deficient in this complication. The grand criterion in either form is the condition of the aggregated glands of Peyer.

The propriety of the division of continued fever into two species, or varieties, and applying to express the distinction the terms typhus and typhoid, we do not purpose to discuss except in so far as the follicular affection is involved, That such a division is convenient, and admissible, *based upon symptoms*, we do not doubt, but that the presence, or absence of the follicular affection holds good, as a general law, in the two divisions, facts have disproved. We will notice the latter sufficiently to establish the disproval, and, afterward endeavor to account for the facts from which the general laws have been erroneously deduced, and consider some sources of fallacy attending these facts. The topic is not one of pathological interest only: it is important, because, if the state of the intestinal canal does afford sure evidence in all cases, that, the disease is either typhus or typhoid, the diagnosis from the symptoms during life must be made to conform to this evidence, there being no one symptom which has the same signifiance and constancy. We shall have occasion presently to observe how this natural consequence of adopting the follicular affection as a post-mortem test has led to error.

It is to be remarked that a few of well authenticated cases of typhus fever, so determined by vital symptoms, in which the glands of Peyer were found diseased, are sufficient to antagonize a host of equally well authenticated cases in which these glands were found healthy. The latter are negative, the former positive facts. Negative facts cannot establish a general law in opposition to positive facts, although the former may greatly outnumber the latter, for a general law contains no provision for exceptional instances. The results of Dr. Gerhard's observations in 1836, were certainly extraordinary, but if in three or four cases only with the same *ensemble* of symptoms, the Peyerian glands were found affected, it would of course be necessary to explain the absence of intestinal lesions in the remainder of the cases otherwise than by supposing that these lesions never occur as a complication of that form of fever. Indeed, the single case in which these glands were diseased would have been sufficient to have upset the induction, had there not been previous doubt as to the diagnosis. Now there are abundant disproving positive facts, but, for the reason just given, it is not necessary to cite a large number. M. Landouzy, of Rheims, France, has described an epidemic occurring at the prison in that place, which presented all the *living* characters of true typhus, inclusive of the absence of diarrhœa, and meteorism. Nevertheless in six autopsies, which were made, the characteristic lesions of typhoid fever, as described by Louis, were discovered in each case. Other instances are referred to by Grisolle, [*Pathologie interne*] and several are collected by Dr. Clymer in his edited work on fever, in a section relating to this subject which was published in a former No. of this Journal.* British writers almost universally testify to the presence of these lesions in cases, which, in the *ensemble* of symptoms belonging to true typhus, cannot be distinguished from cases in which these lesions are absent. The following quotation from Dr. Christison on this point is explicit, and comes from awriter whose opportunities for personal knowledge have been extensive. He says:—"The *constitutio dothinenterica*, to speak in nosographical language, has repeatedly appeared and disappeared, as a subordinate, or intercurrent epidemic, in the course of the more general epidemic, typhus, without the great features of that epidemic being altered in any material respect." Without multiplying references, it seems fair to assume it as settled by facts, that cases of fever, in all the phenomena attending its march, presenting the distinguishing characters of typhus, may furnish the same intestinal lesions which are found in cases of typhoid fever. The question, then, resolves itself into this:—Shall cases of fever which are identical in their phenomena during life, be considered identical whether follicular

---

* Grissolle refers to a work by *M. Gaultier de Claubry*, in which the identity of typhus and typhoid fver is established, both by comparison of the symptoms, and community of anatomical characters. Grisolle considers it settled that the two are identical; nevertheless, he regards the observations of Dr. Gerhard and Dr. Shattuck as establishing a form of fever distinct from typhoid fever.

lesions exist, or not; or shall they be divided according to the presence or absence of these lesions, without reference to identity in the living symptoms? This question certainly does not claim serious discussion. It is enough to ask, of what possible utility is a diagnostic test, which can only be determined by dissection! It is not less ridiculous, than unphilosophical, to institute a nosological division upon a single anatomical character, which can only be revealed by the scalpel! The objects of nosology, and the advantages of diagnosis, relate to the living, not to the dead!

It is evident that if this be recognized as an unfailing pathological pointof distinction, all cases of typhoid fever will have it, and all cases of typhus fever will not have it, simply because it is previously agreed to call all cases in which it is present typhoid, and cases in which it is absent typhus! The *petitio principii* of this method of reasoning is sufficiently manifest. Untenable and absurd as this appears when presented as an abstract proposition, there is reason to believe, that, practically, it has not been without considerable influence. With becoming diffidence we would submit, whether the work of Louis, careful and conscientious as is that distinguished pathologist, may not be obnoxious to criticism on this score. He declares that he found the "anatomical characteristic" in all the fatal cases of typhoid fever in which he performed post-mortem examination; but his work contains some observations of what he distinguishes as "simulated cases of typhoid fever." These cases presented during life symptoms analagous to veritable cases of typhoid fever, but they were different in the intestinal lesions, and it is chiefly on the latter account that he regards them as only simulating the disease. Now in deciding that these cases were not cases of typhoid by the post-mortem diagnostic, did not Louis commit the very *petitio-principii* just mentioned? That he did allow his "anatomical characteristic" to furnish the rule for admitting and rejecting cases he distinctly admits, as in the following comments, after the details of one of his observations:—"This fact presents, as we see, the union of almost all the most characteristic symptoms of the typhoid affection. * * * What more was wanting to make us certain that the patient was affected with the typhoid disease, and that we should find, on opening the corpse, the eilliptical patches of the small intestine more or less seriously altered? Notwithstanding this, the patches, and the corresponding merenteric glands, were healthy, &c." Thus it appears, that after finding lesions of Peyer's glands uniformly present in a certain series of cases, Louis adopts these lesions as a diagnostic test, and afterward calls cases in which this test fails, "simulated cases of typhoid affection." Conceding that this course was unphilosophical, it follows that the latter were probably cases of veritable typhoid; and, hence, the incorrectness of his generalization is proven from his own facts, as it has been abundantly proven by facts observed by others since the publication of his work.

Again, Louis presents observations of "latent typhoid fever," in which the characteristic symptoms were absent, but the follicular lesions were found after death. Without dwelling upon the subject, we would simply remark, that the same criticism is applicable here, as in the former instances. And if the criticism be valid, the conclusion of Louis that these lesions are not found after death from other acute diseases, is equally disproved by his own facts, as it has been also disproved by the observations of others. Dr. Lombard, following Louis in the adoption of the intestinal lesions as a diagnostic sign sufficient in itself to determine the fact of typhoid fever, has reported cases in which patients exhibited none of the ordinary symptoms of fever up to their disease, in one instance death occurring by self-destruction, nevertheless, these lesions being found on dissection, he considers them cases of "latent typhoid." [*Etudes cliniques sur quelques points de l'histoire des Fievres Typhoides por les Docteurs Lombard et Fairconsies*]. Surely it is not presumptious to say that the supposition of the existence of fever under such circumstances is quite gratuitous.

If these criticisms were only interesting as relating to the works named, an apology would be necessary for presenting them, more especially as the publication of Louis has been before the profession some fourteen years. But the errors referred to, are, to some extent, still operative. Hence, it seems to us important to establish the position that nosological sub-divisions of continued fevers should be based mainly on differences in the phenomena manifested during life, and that the presence or absence of follicular lesions is of secondary consequence. Diagnostic traits should be founded on circumstances pertaining to symptoms, etiology, and anatomical characters collectively. A particular circumstance belonging to the latter, can no more be considered a criterion than a particular symptom, and either adopted as an exclusive diagnostic, cannot fail to lead to the disunion of cases which are essentially similar, and to the union of cases which are dissimilar. Continued fevers are doubtless susceptible of being nosologically sub-divided, but this subject we do not purpose now to consider. Our object is only to show that the presence or absence of follicular enteritis is not to be relied on as adequate to distinguish a particular species or variety, nor even continued fever without regard to its sub-divisions.

We come now to inquire how we are to reconcile in the first place the fact that Louis found follicular lesions in forty-eight conventive autopsies, and, next, the fact that Dr. Gerhard found in fifty autopsies no such lesions, with other facts which have demonstrated that in the so called typhoid fever, these lesions may be absent, and in the so called typhus fever they may be present. The most rational explanation is one which is not original with us, viz., that in the first instance the presence of these lesions constituted a peculiarity of the disease (i. e., continued fever), at the locality, and during the period it was studied by Louis; and, in the latter instance, the absence of these lesions was a peculiar feature of the disease at the time and place it was studied by Dr. Gerhard. That epidemics of different seasons, and regions, exhibit different phases, presenting sometimes certain peculiarities common to all cases at that period, which are as universally absent at another period, is by no means a new truth in pyretology. It is, at least, as old as Sydenham, who enjoined the importance of studying closely the "constitution of the year," with reference to these peculiarities. The *constitutio dothinenterica*, which Dr. Christian states has prevailed as an intercurrent affection at different times in Edinburg, characterized the Parisian fevers during the time Louis recorded his observations; and its absence was equally characteristic of the Philadelphia epidemic of 1836. We have seen that among the cases of Louis were probably veritable cases of typhoid fever in which the follicular lesions were wanting, which he rejected and distinguished as cases simulating the disease. The latter would doubtless have been much more numerous had he continued to collect cases for a longer period: and, so, another importation from Liverpool to Philadelphia of veritable typhus might have furnished results quite different from those distinguishing the epidemic investigated by Dr. Gerhard. Other circumstances may have more or less influence in determining the complication under consideration. English writers are fond of attributing its greater apparent prevalence in France to the difference in dietetic habits of the two nations, and the difference of treatment, especially as concerns purgative medicines, which are less employed in the treatment of fever in France, than in England. We have no data, however, for attributing the complication to any specific agencies. Whatever these agencies are (and they doubtless exist, for this, not less than other peculiarities, cannot be purely accidental) observations continued for a long period are necessary for their discovery or demonstration.

By the foregoing remarks we would not be thought to undervalue the investigations of Louis, and others, with respect to the follicular complication of fever. Although we contend that in so far as it is considered distinguishing exclusively different species, or varieties of fever, it is calculated to lead to serious errors, we do by no means deny, nor would we depreciate the service which has been rendered to science by conferring a better knowledge of its frequency and character; and this has resulted from late researches. It is to be hoped that continued investigations will shed more light on the nature and extent of its pathological relations. It were much to be desired that we possessed more accurate information respecting its occurrence in miasmatic and eruptive fevers, as well as in other maladies. The accumulation of facts may hereafter, not only enable us to determine more accurately its value as an element of fever, but to understand the laws by which it is governed.

The possibility, or probability of this complication, should not be lost sight of by the Practitioner in the treatment of fever. Although it is by no means certain that it exerts much, if any controling influence over the duration of the disease, or over the phenomena accompanying its march, except those which have with it a palpable connection, yet, obviously, its therapeutical relations are not unimportant. Drastic purgatives, on the one hand, and over accumula tion of the intestinal contents, on the other hand, must exert an injurious effect upon the diseased follicles. The fact that when fever terminates favorably the enteritic lesions are often not fully restored, enforces the importance of great care, as respects diet, &c., for some time after convalescence established.

The practitioner should be prepared to meet occasionally with cases of perforation from progressive ulceration through the coats of the intestine, followed by fatal peritonitis, It has so happened that two cases have fallen under our observation within a few weeks, The liability to this accident renders it prudent to be guarded in our prognosis in cases of suspected enteritic complication, even when the general symptoms are favorable, for it is found to occur quite as often in mild, as in severe cases, and, as already stated, may take place during convalescence, after all fear of danger in the minds of patients and friends has passed away.

